# Management of meige syndrome with bilateral trigeminal and facial nerves combing

**DOI:** 10.3389/fneur.2025.1626729

**Published:** 2025-07-29

**Authors:** Huiling Peng, Xue Li, Bing Huang

**Affiliations:** ^1^Graduate School of Zhejiang University of Traditional Chinese Medicine, Hangzhou, Zhejiang, China; ^2^Pain Management Center, Department of Anesthesiology, The Second Affiliated Hospital, Zhejiang University School of Medicine, Hangzhou, Zhejiang, China

**Keywords:** cranial nerves combing, meige syndrome, radiofrequency ablation, facial nerve, trigeminal nerve

The recent publication by Li et al. (1) detailing novel neurosurgical intervention through bilateral trigemino-facial nerve combing presents a paradigm-shifting approach in Meige Syndrome (MS) management, with preliminary outcomes underscoring its therapeutic potential.

Since its first description and eponymous designation by French neurologist Henry Meige in 1910 (2), the precise pathogenesis of MS has remained elusive, therapeutic challenges that have persisted for over a century. Current clinical management faces three principal limitations: the absence of effective oral pharmacotherapy; the transient efficacy of botulinum toxin injections complicated by antibody development with repeated administrations; and the suboptimal cost-benefit ratio of deep brain stimulation (DBS) given its prohibitive expense relative to clinical efficacy. While facial neurectomy has demonstrated symptomatic relief in limited reports, the procedure's clinical application has been abandoned due to consequent severe iatrogenic facial paralysis (2).

Professor Li's team has developed an innovative nerve root combing technique targeting both facial and trigeminal nerves, demonstrating clinically meaningful symptom alleviation in Meige Syndrome (MS) with favorable patient-reported outcomes (1). This approach marks a substantial refinement over conventional neuroablative procedures through its implementation of selective neural modulation. Unlike facial neurectomy that risks complete functional impairment, the combing technique achieves partial disruption of nerve fibers while preserving critical neural continuity, thereby maintaining baseline orofacial motor function and preventing severe iatrogenic complications. Generally speaking, the motor signals of voluntary movements (movements controlled by subjective consciousness) are regulated by the brain, and the signal strength is much greater than the strength of the ectopic motor signals that cause involuntary movements. As long as part of the motor nerve's conduction ability is blocked, the ectopic motor signals may be filtered out, thus eliminating the involuntary movements, and the stronger signal of the random movements may still be transmitted down to the effector muscles while retaining part of the random motor function (3).

Building upon this neurophysiological rationale, our team has implemented CT-guided radiofrequency ablation (RFA) targeting the extracranial segments of culprit cranial nerves, demonstrating significant therapeutic efficacy across MS subtypes (3). MS is pathophysiologically characterized as a medially symmetric segmented craniocervical dystonia involving single or multiple cranial nerve groups (4, 5). The clinical manifestations exhibit precise anatomical correlation: facial nerve involvement manifests as bilateral spasms in mimetic muscles (frontalis, orbicularis oculi, orbicularis oris, platysma), trigeminal nerve dysfunction presents with masticatory muscle hypertonia, accessory nerve pathology correlates with spasmodic torticollis, hypoglossal nerve compromise induces lingual dyskinesia, multineuronal involvement generates complex phenotypic combinations. Our clinical protocol mandates bilateral extracranial RFA for all implicated neural pathways (4–6). Therapeutic strategy is individualized based on MS classification. Minimum intervention requires bilateral RFA of one cranial nerve group. Composite forms necessitate sequential ablation of more than 2 nerve groups to achieve comprehensive symptom resolution (5).

The neurosurgical intervention “Bilateral Trigeminal and Facial Nerves Combing for Meige Syndrome Management” demonstrates significant efficacy in alleviating spastic symptoms of the composite neurotype (blepharospasm-plus oromandibular dyskinesia subtype) involving facial and trigeminal nerve pathology (1). However, this dual nerve intervention remains unwarranted for isolated facial or trigeminal nerve subtypes. Notably, in complex multineuronal MS presentations, even combined nerve root combing proves insufficient for complete symptom resolution, necessitating adjunctive modulation of additional cranial nerve groups. Furthermore, for postoperative recurrence cases, extracranial RFA therapy represents a more viable retreatment option compared to repeat craniotomy procedures.

Preliminary evidence suggests that extracranial RFA therapy may present a more favorable safety profile and cost-effectiveness ratio compared to intracranial nerves combing procedures requiring craniotomy. In our study of 58 MS patients, the main complication is recoverable mild to moderate facial paralysis, with a 100% effectiveness rate in reducing blepharospasm. The RFA procedure duration is 30.79 ± 7.69 min, and the cost of hospitalization is 8,997.94 ± 1,269.02, compared to 149,331.92 ± 1,987.23 in MS patients who underwent DBS (4).This approach appears to offer clinical advantages particularly in terms of reduced procedural risks and healthcare resource utilization.
